# Measuring the bias against low-income country research: an Implicit Association Test

**DOI:** 10.1186/s12992-017-0304-y

**Published:** 2017-11-06

**Authors:** Matthew Harris, James Macinko, Geronimo Jimenez, Pricila Mullachery

**Affiliations:** 1 Institute of Global Health Innovation, Imperial College London, 10th Floor, QEQM building, St. Mary’s Campus, Praed Street, London, W2 1NY England; 2 UCLA Fielding School of Public Health, Center for Health Sciences, 650 Charles E. Young Dr. South, Room 31-235B, Los Angeles, CA 90095-1772 USA; 3 Centre for Population Health Sciences (CePHaS), Lee Kong Chian School of Medicine, Nanyang Technological University, 11 Mandalay Road Level 18 Clinical Sciences Building, Novena Campus, 308232 Singapore, Singapore; 4 NYU College of Global Public Health, 726 Broadway, New York, NY 10012 USA

**Keywords:** Implicit association test, Bias, Research evaluation, Stereotypes, Reverse innovation

## Abstract

**Background:**

With an increasing array of innovations and research emerging from low-income countries there is a growing recognition that even high-income countries could learn from these contexts. It is well known that the source of a product influences perception of that product, but little research has examined whether this applies also in evidence-based medicine and decision-making. In order to examine likely barriers to learning from low-income countries, this study uses established methods in cognitive psychology to explore whether healthcare professionals and researchers implicitly associate good research with rich countries more so than with poor countries.

**Methods:**

Computer-based Implicit Association Test (IAT) distributed to healthcare professionals and researchers. Stimuli representing Rich Countries were chosen from OECD members in the top ten (>$36,000 per capita) World Bank rankings and Poor Countries were chosen from the bottom thirty (<$1000 per capita) countries by GDP per capita, in both cases giving attention to regional representation. Stimuli representing Research were descriptors of the motivation (objective/biased), value (useful/worthless), clarity (precise/vague), process (transparent/dishonest), and trustworthiness (credible/unreliable) of research. IAT results are presented as a Cohen’s d statistic. Quantile regression was used to assess the contribution of covariates (e.g. age, sex, country of origin) to different values of IAT responses that correspond to different levels of implicit bias. Poisson regression was used to model dichotomized responses to the explicit bias item.

**Results:**

Three hundred twenty one tests were completed in a four-week period between March and April 2015. The mean Implicit Association Test result (a standardized mean relative latency between congruent and non-congruent categories) for the sample was 0.57 (95% CI 0.52 to 0.61) indicating that on average our sample exhibited moderately strong implicit associations between Rich Countries and Good Research. People over 40 years of age were less likely to exhibit pro-poor implicit associations, and being a peer reviewer contributes to a more pro-poor association.

**Conclusions:**

The majority of our participants associate Good Research with Rich Countries, compared to Poor Countries. Implicit associations such as these might disfavor research from poor countries in research evaluation, evidence-based medicine and diffusion of innovations.

## Background

Research in the marketing field suggests that references to a product’s country of origin (source country) can evoke either positive or negative consumer stereotypes [1–4]. Source country name can be used as a proxy for product quality^4^ resulting in biased judgments. Consider for example “Italian ice-cream”, “German engineering” and “Swiss watches”. Bilkey and Nes [5] showed that consumers tend to rate products from their own countries more favorably and that consumer preferences are positively correlated with the degree of economic development of the source country, probably evoked by the lower price cue of low-income country products [5]. Up to 30% of the variance of consumer product ratings can be attributed to the product’s country-of-origin [6].

Whereas in the marketing industry this extrinsic product cue is communicated by the phrase “made in….” [6], in healthcare research it is perhaps equally communicated by the phrase “author’s affiliation”. Publication rates certainly seem to be related to the income and development level of the source country, [7–16] however there is a need for a more detailed empirical examination of how source country impacts decision-making processes and evaluation of research, from the likelihood of positive peer-review to post-publication credibility of results. In a recent study, Harris et al. [17] show that, in some circumstances, US public health researchers evaluate research abstracts less favorably when the source is from a low-income country. In a randomized, blinded, cross-over study, Harris et al. (2017) show that English clinicians rate abstracts from low-income countries less favourably, controlling for the individual rater, and the type of research [18]. There was a significant overall impact of high-income country source on respondents’ ratings of relevance (4.50, 3.16 to 5.83, *p* < 0.001) and recommendation to a peer (3.05, 1.77 to 4.33, *p* < 0.001). Although a small difference, this measurable, statistically significant difference may be ‘clinically’ important at scale, considering the quantity of research consumed globally. In a related study, Harris et al. [19] found that translating innovations from low-income countries into the US health system can be thwarted early on by perceptions that such countries are incapable of offering learning of any value to the US (‘*They hear “*Africa*” and think that there can’t be any good services*’) [19] and that adopting innovations from low-income countries is somewhat ‘non-traditional’ [20] (‘*That’s not how the learning works*’).

From a methodological standpoint, it is challenging to ascertain why source impacts on peoples’ perceptions of a research article, because, even under experimental conditions, attitudes to certain country sources will be either consciously unavailable or mediated by social desirability bias [21]. Empirical studies from the marketing literature that examine the effect of country-of-origin have predominantly used rating scales to measure respondents’ view of the product. Some techniques are available to reduce social desirability bias, such as maintaining anonymity, indirect questioning, [22] social desirability scales [23] and randomized response [24]. However, questions on prejudice or stereotype may not be accessible to rational thought. This information can be unavailable to introspection even if there is motivation to reveal it – much like memories [25]. There is now an extensive literature revealing, empirically, associative information that people are either unwilling or unable to report [21]. Techniques have emerged to measure attitudes inaccessible to introspection, such as the Go/No-Go Association Task [25] and the Extrinsic Affective Simon Task (EAST) used in the management of phobias and anxiety [26, 27].

The Implicit Association Test [28] has been shown to demonstrate superior performance in detecting biases [21]. The IAT measures the relative latencies (time taken to respond) between associative sorting tasks. Respondents click one of two computer keys to categorize stimuli into associated categories. When the categories seem consistent to the respondent, the time taken to categorize the stimuli will be less than when the categories seem inconsistent. An implicit association is said to exist when respondents take longer to respond to a category-inconsistent pairing than a category-consistent pairing. Sorting is easier, and therefore quicker, when two concepts are strongly associated than when they are weakly associated [21]. The test provides a palpable experience of the greater ease of some associations and the relative uncontrollability of such associations [29]. As a result it provides a more direct experience of potential dissociations between one’s conscious and unconscious attitudes and beliefs [21].

In the social psychology literature the IAT has been applied in a number of settings, for example to measure the association between gender and science subjects, and gender and careers (see Nosek et al. [21] for a full review). In the marketing literature, Martin et al. [4] used the IAT to determine whether exposure to a country of origin cue will result in automatic activation of stereotypes to evaluate certain products. Based on the premise that the process of evaluating research may be subject to some of the same perception biases as have been found in other fields, we use the IAT methodology to ascertain whether respondents, healthcare researchers and practitioners, associate good research with either high-income countries or low-income countries.

## Methods

### IAT design

Detailed appraisal of the internal validity of the IAT method, particularly the internal consistency, test-retest reliability and fakeability of the IAT is discussed in detail in Nosek et al. [21]. Our IAT survey was split in to three sections – demographic information, the IAT test and an explicit association survey question. We collected data on year of birth, gender, education attainment, country of birth and where the respondent currently lives, exposure to research articles, whether a peer reviewer or not and what kind of organization the respondent works in.

Critical elements of the IAT are to ensure that the stimuli items are clear, used for the intended categorization, and not for an alternative stimulus feature. IAT stimuli can be words, pictures, sounds or combinations [21]. The IAT section in this study contained four categories – rich country/poor country and good research/bad research. Country stimuli were selected from the World Bank 2013 rankings for GDP per capita (World Bank 2013 http://data.worldbank.org/indicator/NY.GDP.PCAP.CD). Rich countries were chosen from OECD members in the top ten (>$36,000 per capita) rankings and poor countries were chosen from the bottom thirty (<$1000 per capita) countries by GDP per capita, in both cases giving attention to regional representation. Stimuli to represent research are less clear-cut. Research can be taken to mean research question, research paper, research methods, research presentation or even researcher. Characteristics of good qualitative research are likely to be different also to quantitative research as both require different attributes and skills and have different types of outputs. We tried to determine some enduring features common to the scientific method, irrespective of the type of research, minimizing conceptual overlap between the features whilst maximizing the types of features included. Consensus was generated through several iterations within the research team and consultation with peers. Stimuli chosen represent descriptors of the motivation (objective/biased), value (useful/worthless), clarity (precise/vague), process (transparent/dishonest), and trustworthiness (credible/unreliable) of research.

The category stimuli are listed in Table 1. The IAT was organized into seven blocks (Table 2). Order effect, when the performance of a task is affected by a preceding task [30] was overcome by randomizing the order of blocks 3/4 and 6/7 for each participant, in accordance with the approach described by Nosek et al. [21]

**Table 1:** Categories and stimuli used in the IAT

Rich country	Poor country	Good research	Bad research
Canada	Malawi	Objective	Biased
United Kingdom	Ethiopia	Precise	Vague
Japan	Cambodia	Transparent	Dishonest
Germany	Liberia	Credible	Unreliable
France	Bangladesh	Useful	Worthless

**Table 2:** Sequence of Blocks in the IAT

Block	No. of trials	Items assigned to left-key	Items assigned to right-key
B1	20	Poor countries	Rich countries
B2	20	Good research	Bad research
B3	20	Poor countries + Good research	Rich countries + Bad research
B4	40	Poor countries + Good research	Rich countries + Bad research
B5	40	Rich countries	Poor countries
B6	20	Rich countries + Good research	Poor countries + Bad research
B7	40	Rich countries + Good research	Poor countries + Bad research

(adapted from Nosek et al. [18])

We also included an explicit survey question (“Do you think that Poor Countries are as likely as Rich Countries to produce Good Research”) on a five-point Likert scale response (Strongly agree vs Strongly Disagree). We randomized the order of the explicit survey question with the IAT test itself, as recommended by Nosek et al., [21] because the order may increase the accessibility of some cognitive functions and influence self-report.

### Survey administration

IAT surveys have been used either under laboratory conditions [4] or for general use by the public, for example at Internet kiosks in a museum [29] and more broadly still via the Project Implicit website where a variety of IAT tests are available to be taken by anyone with an interest in understanding their own hidden associative biases. We were interested to capture, as far as possible, the IAT scores of those individuals to whom this particular research question will be most relevant i.e. healthcare researchers, managers and policy makers. We distributed the link to the survey through the Academy Health membership network, a network of academics, managers and policy makers within the US healthcare landscape. The survey link was first distributed on the 26th March 2015 through a general email list, then again as a single-purpose email to a random sample of 40% and then as a Tweeted link to the survey. On the 31st March 2015 we then distributed the link to the survey through NYU ListServes that include students and faculty from a variety of health, nutrition, public health, medicine and social policy backgrounds. We posted the link to the survey on specialist interest group websites, such as the American Association for Tropical Medicine and Health, the Consortium of Universities in Global Health and LinkedIn Global Health.

We collected data for just 4 weeks. No other reminders were sent and there were no inducements or incentives to take the survey. Participants were presented with an introduction screen that included a Consent Agreement stating that participation was voluntary, non-compensated and with no risk. The Consent Agreement also stated that responses were confidential and anonymous, protected and analyzed in the aggregate, and with information of who to contact in the event of any queries. Also included was the Project Implicit privacy policy. Consent was presumed through participation in the survey. The protocol for the research was reviewed by the University Committee for Activities Involving Human Subjects at New York University and deemed exempt from full review (IRB #14–10,332).

### IAT analysis

Greenwald et al. [31] recommend using the D algorithm to present IAT responses because it exhibits the greatest internal validity of all the algorithms so far developed by the Project Implicit research team, it is relatively uninfluenced by IAT experience or small sample size, and it reduces the influence of cognitive fluency, which tends to be greater with age [32]. Following Nosek et al. [29] we report IAT effects as a Cohen’s d (standardized mean) for the whole sample.

The top two categories of the explicit survey question were coded as ‘agree’ with all other responses as the reference. Predictors of the explicit survey question are determined through stepwise inclusion of socio-demographic (age/sex), birth/residence, and academic experience and employment location independent variables using Poisson multivariable regression models. Robust standard errors are used because of evidence of heteroskedasticity.

We identified the range in the IAT response distribution that corresponded to each of the standard IAT categories that define different levels of implicit association. We chose the midpoint of each of these ranges and performed quantile regression to understand the contribution of our explanatory variables to each of these different categories of response. Quantile regression (as opposed to ordinary least squares (OLS) regression which estimates the conditional mean of the outcome variable) estimates the conditional distribution of the response variable at its median or other percentile of its distribution. Results are presented as regression coefficients with robust 95% confidence intervals and interpreted analogous to those derived from OLS. These categories and corresponding IAT values are as follows: −.35 to −.15, slight association between Good Research and Poor Countries; −.15 to +.15, little to no association; +.15 to +.35, slight association between Good Research & Rich Countries; +.35 to +.65, moderate association between Good Research & Rich Countries; +.65 and higher, strong association between Good Research & Rich Countries. These IAT categories correspond to the 4th, 12th, 23rd, 45th, 60th centiles in our distribution. We also examined the extreme right-hand side of our distribution and included the 90th centile in our distribution as a very strong association between Good Research and Rich Countries. Univariate, Poisson and Quantile regression analysis was conducted using Stata/SE 14 (Statacorp, College Station, Texas).

### Participants

The sample of 321 individual tests was predominantly female, below the age of 40 and had primarily academic qualifications (Table 3). More than half were born outside the US, however more than half currently reside in the US. 55% of the sample had been born and currently live in the US. Two-thirds of the sample works at a University, however less than 50% peer review for an academic journal and read a research article on a daily basis.

**Table 3:** Respondent characteristics, IAT effect and explicit survey measures

			IAT	Explicit survey	
Independent variables	N	%	Cohen’s d (95% CI)	% Agree	% Not agree
Males	83	25.86	0.61 (0.53, 0.69)	25.61	74.39**
Females	225	70.09	0.55 (0.50, 0.60)	48.42	51.58
20–29	81	25.23	0.54 (0.46, 0.63)	37.50	62.50
30–39	119	37.07	0.53 (0.46, 0.59)	41.18	58.82
40–49	60	18.69	0.59 (0.50, 0.69)	45.76	54.24
50–59	34	10.59	0.62 (0.49, 0.75)	40.62	59.38
60+	16	4.98	0.75 (0.61, 0.89)	56.25	43.75
Academic qualification only	276	85.98	0.55 (0.51, 0.60)	42.28	57.72
Clinical qualification only	16	4.98	0.60 (0.42, 0.78)	43.75	56.25
Clinical-Academic qualification	18	5.61	0.69 (0.52, 0.87)	55.56	44.44
US born	146	46.79	0.57 (0.51, 0.63)	45.07	54.93
Non-US born	163	52.24	0.57 (0.51, 0.63)	38.27	61.73
US lives	180	57.32	0.56 (0.51, 0.62)	48.59	51.41
Non-US lives	127	40.45	0.57 (0.51, 0.63)	33.33	66.67
Live and born in US	176	54.83	0.56 (0.50, 0.62)	40.23	59.77*
Live and born in different countries	138	42.99	0.58 (0.52, 0.64)	45.93	54.07
Works at a University	211	66.56	0.56 (0.51, 0.61)	39.42	60.58
Does not work at a University	106	33.44	0.58 (0.51, 0.64)	48.08	51.92
Reads research daily	146	45.91	0.56 (0.50, 0.63)	41.78	58.22
Reads research less than daily	172	54.09	0.57 (0.51, 0.63)	43.11	56.89
Peer reviewer	173	45.17	0.53 (0.47, 0.59)	39.77	60.23
Not a peer reviewer	145	53.89	0.61 (0.55, 0.66)	45.77	54.23
IAT response categories					
Moderate pro-poor	4	1.25	−0.509 (−0.57, −0.45)	100.00	0.00
Slight pro-poor	8	2.49	−0.232 (−0.28, −0.19)	37.50	62.50
Neutral	32	9.97	0.017 (−0.02, 0.05)	34.38	65.62
Slight pro-rich	43	13.4	0.251 (0.232, 0.27)	54.76	45.24
Moderate pro-rich	94	29.28	0.515 (0.50, 0.53)	39.78	60.22
Strong pro-rich	67	20.87	0.748 (0.74, 0.76)	47.69	52.31
Very strong pro-rich	73	22.74	1.038 (1.00, 1.07)	34.72	65.28
Total	321	100.00	0.57 (0.52, 0.61)	42.41	57.59

*p < 0.05 **p < 0.01

## Results

The mean IAT score was 0.57 (95% CI 0.52 to 0.61) indicating that on average our sample exhibited moderately strong implicit associations between Rich Countries and Good Research (Table 3). This is reflected in the response to the explicit survey question – fewer people (42.41%) agreed that Poor Countries are as likely as Rich Countries to produce Good Research (57.59% did not agree). Males were significantly more likely to disagree with the explicit survey question compared to females (74.39% vs 51.58%) and people that were born and live in the US were significantly more likely to disagree with the explicit survey question compared to people that were born and live in different countries (59.77% vs 54.07%).

There was no statistically significant difference in IAT scores between participants who agreed with the explicit survey question (0.59, 95% CI 0.53 to 0.64) and those who disagreed with it (0.53, 95% CI 0.47 to 0.59) – people with pro-Poor or pro-Rich Country implicit associations were equally likely to agree or disagree with the explicit survey question. Multivariable Poisson regression of explicit survey responses however showed that Females and individuals over 60 years of age were more likely to agree with the explicit survey question (IRR 2.03 (1.36, 3.02 *p* < 0.001) and 1.79 (1.12, 2.84 *p* < 0.05) respectively) (Table 4) compared to the reference categories.

**Table 4:** Predictors of agreeing/strongly agreeing to the statement, “Poor countries are as likely as rich countries to produce good research”

Independent variable	IRR
Female (v male)	2.03 (1.36, 3.02)***
30–39 (v 20–29)	1.15 (0.95, 4.36)
40–49 (v 20–29)	1.25 (0.80, 1.67)
50–59 (v 20–29)	1.13 (0.67, 1.89)
60+ (v 20–29)	1.79 (1.12, 2.84)*
Clinical (v academic)	1.19 (0.68, 2.07)
Live and birth in US (v different)	0.95 (0.74, 1.22)
Works at a university (v elsewhere)	0.86 (0.64, 1.16)
Peer review (v not)	0.87 (0.65, 1.17)
Reads research daily (v less than daily)	1.00 (0.76, 1.31)
N	313

*p < 0.05 ***p < 0.001

Coefficients representing missing values are not represented in the table

Table 5 displays results from the quantile regressions. Column 1 shows that for the slightly pro-poor portion of the IAT distribution the coefficients on ages 40 and over are positive, and statistically significant, as compared to the reference group (40–49 years - 0.29 (0.09,0.49 *p* < 0.01); 50–59 years – 0.44 (0.15, 0.73 *p* < 0.01); 60 years and above – 0.55 (0.20, 0.90 *p* < 0.01). We note however that this positive association should be interpreted negatively given that the IAT values at the 4th percentile (slightly pro-poor) are below zero. That is, people at older ages are less likely to fall into the pro-poor category. For the regressions predicting no association and slightly pro-rich associations (columns 2 and 3) only older ages (40 and above) had a positive association in each model (No association 0.33 (0.99, 0.57 *p* < 0.01); Slightly pro-Rich 0.29 (0.06, 0.45 *p* < 0.01). Table 5 also shows that peer review was negatively associated with each outcome as compared to people that do not peer review, suggesting that peer review contributes to a more pro-poor association (noting the need to inverse the interpretation for the coefficients presented in column 1) (Slightly pro-Poor −0.2 (−0.31, −0.09 *p* < 0.001); No association −0.27 (−0.39, −0.14 *p* < 0.001); Slightly pro-Rich −0.19 (−0.32, −0.07 *p* < 0.001); Moderately pro-Rich −0.14 (−0.23, −0.05 *p* < 0.001).

**Table 5:** Quantile regression coefficients of IAT score

	Slightly pro-poor	No association	Slightly pro-rich	Moderate pro-rich	Strong pro-rich	Very strong pro-rich
Female (v male)	−0.13	−0.06	0.07	0.01	−0.07	−0.1
	(−0.26,0.00)	(−0.17,0.06)	(−0.09,0.23)	(−0.09,0.11)	(−0.20,0.07)	(−0.21,0.00)
30–39 (v 20–29)	0.19*	0.09	0.13	−0.04	−0.01	0.07
	(0.02,0.35)	(−0.06,0.23)	(−0.09,0.35)	(−0.18,0.10)	(−0.14,0.12)	(−0.07,0.20)
40–49 (v 20–29)	0.29**	0.33**	0.26**	0.05	0.02	0.23**
	(0.09,0.49)	(0.09,0.57)	(0.06,0.45)	(−0.09,0.20)	(−0.15,0.19)	(0.08,0.37)
50–59 (v 20–29)	0.44**	0.42***	0.26*	0.13	0.03	0.08
	(0.15,0.73)	(0.27,0.57)	(0.05,0.48)	(−0.02,0.28)	(−0.13,0.19)	(−0.08,0.24)
60+ (v 20–29)	0.55**	0.45***	0.41**	0.19	0.05	0.22
	(0.20,0.90)	(0.23,0.66)	(0.16,0.65)	(−0.01,0.40)	(−0.19,0.29)	(−0.18,0.62)
Clinical (v academic)	−0.06	0.07	0.26	0.16*	0.05	−0.17*
	(−0.70,0.59)	(−0.09,0.22)	(−0.05,0.57)	(0.04,0.28)	(−0.10,0.20)	(−0.33,-0.00)
Clinical academic (v academic)	0.16	−0.01	0.19	0.17***	0.09	0.09
	(−0.65,0.98)	(−0.18,0.16)	(−0.22,0.61)	(0.07,0.27)	(−0.05,0.23)	(−0.11,0.29)
Live and birth in US (v different)	−0.04	−0.09	−0.05	−0.02	−0.04	−0.05
	(−0.16,0.07)	(−0.21,0.02)	(−0.17,0.08)	(−0.12,0.07)	(−0.15,0.07)	(−0.16,0.05)
Peer review (v not)	−0.2***	−0.27***	−0.19**	−0.14**	−0.11	−0.05
	(−0.31,-0.09)	(−0.39,-0.14)	(−0.32,-0.07)	(−0.23,-0.05)	(−0.24,0.01)	(−0.15,0.04)
Reads research daily (v less than daily)	0.06	0.09	0.03	0.07	0.1	−0.08
	(−0.07,0.19)	(−0.02,0.21)	(−0.08,0.14)	(−0.03,0.16)	(−0.01,0.21)	(−0.17,0.02)
Works at a university (v elsewhere)	0.01	−0.02	0.04	0.03	−0.02	0.06
	(−0.29,0.30)	(−0.13,0.09)	(−0.10,0.18)	(−0.08,0.14)	(−0.13,0.09)	(−0.03,0.16)
Constant	−0.16	0.13	0.18	0.55***	0.77***	1.09***
	(−0.49,0.17)	(−0.01,0.28)	(−0.06,0.42)	(0.38,0.72)	(0.60,0.94)	(0.92,1.25)
N	309	309	309	309	309	309

*p < 0.05 **p < 0.01 ***p < 0.001

Results are coefficients with robust 95% confidence intervals

For the moderately pro-rich part of the IAT distribution (column 4), peer review continues to be significant and negative, and clinical qualification, as compared to academic qualification only, are positive and statistically significant. In the strong pro-rich model (column 5) no co-variates were statistically significant. Only in the very strong pro-rich parts of the IAT distribution (column 6), was the age group 40–49 positive, while having a clinical qualification was negatively associated with this outcome.

## Discussion

This research found that, on average, our sample of respondents exhibited moderately strong association between Rich Countries and Good Research, and Bad Research and Poor Countries (mean IAT 0.57). Although it is difficult to compare with other IATs, this result is of a similar order to tests of implicit association between male gender and science (0.72), male gender and careers (0.72), and Black-sounding names and negative valence (0.88) [29]. Over 80% of our sample had a slight to strong implicit association between Rich Countries and Good Research. Older individuals were more likely to associate Good Research with Rich Countries, and peer reviewers were more likely to associate Good Research with Poor Countries. Having a clinical qualification was related to associating Good Research with Rich Countries in some circumstances. We found that other individual characteristics, such as where the respondent was born and lives, their gender, the exposure to research and the type of organization they work in, had no contribution to the distribution of IAT scores.

Although we found that women and older individuals were more likely to agree with the explicit survey question, we also found IAT scores were not correlated with the responses to the explicit survey question. This may indicate some level of social desirability bias in the explicit survey question, however differences in response to explicit and implicit tests do not suggest that one is accurate (or real) and one is not. Rather, they suggest a form of mental (and often unrecognized) dissociation between implicit and explicit feelings and thoughts [29]. Implicit and Explicit assessments have separate predictive utility [33]. The IAT is an instrument to help the individual become aware of associations that even through introspection they might not have been aware of and helps us to reflect on the extent to which stereotypes may be applied in our everyday practice [21]. Aggregated mean IAT scores, although small, can have significant societal impact for two reasons – they can explain discriminatory impacts that affect many people simultaneously or they can repeatedly affect single persons [34].

There has been a long-standing and important debate about the validity of the IAT methodology, in particular whether it can be explained by a clear psychometric theory, whether a difference score at the observed level has empirical justification, whether a zero value has meaning and the relative merits of a single or double construct IAT [35]. The exact origin of all forms of attitude and beliefs are not known [31]. Furthermore, it is not entirely clear whether the IAT effects are due to the individual features of the stimulus items, or attitudes towards the categories [21]. If we had chosen different stimulus items then we may have found a different response in some people and we cannot be sure that for some people the stimulus items are representing the intended categories. This issue is, however, a problem with any IAT. For example, in IATs looking at attitudes to race, it cannot be assumed that by looking at a picture of a face it is the skin colour that is being assessed or the shape of the person’s face or size of the nose.

As De Houwer notes (2002) [36], there is a possibility that an IAT effect is dependent on the stimuli that were used, which in this study would mean that the negative stimuli we used were conceptually associated with Poor Countries. We chose descriptors of Research, and although these stimuli are not unequivocally only descriptors of Research, they are valid stimuli because they are not inherently linked to the income level of a country. A Poor Country is not Vague, just as a Rich Country is not Precise, for example. Stimuli, in specific conditions, can play a more critical role than the categories in the IAT effect and can even change the classic IAT effects generally driven by those specific categories [37]. However others argue that categories are more important than the stimuli [36].

In this study, we were interested to understand whether respondents view research from different types of countries as trustworthy. This positive or negative valence could be capturing some general ‘how trustworthy is this country’ association, but there is no reason that this would not also relate to perceptions of their research. Further research could explore whether respondents associate certain countries with Research, removing the valence issue. In this type of study, the IAT could use research-related words as stimuli (e.g., science, experiment and laboratory) in a single-category IAT in which poor and rich countries are paired only with a target category (i.e., Research). In this case, in one block participants will classify “Poor countries” (e.g., Malawi, Cambodia and Liberia) and “Research” (e.g., science, experiment and laboratory) stimuli by pressing a response key, while they will categorize “Rich countries” (e.g., Canada, France and Germany) with another response key. In another block, participants will use a response key to classify “Poor countries”, and the other response key for “Rich countries” and “Research” stimuli. In this way, authors can assess how the concept research is associated with poor/rich countries. If participants are faster in the latter than in the former block, then it suggests that there is a stronger association between “Rich countries” and “Research”.

In our study, as with any IAT, we did not disguise the purpose and objective of the IAT, and subjects do report being aware of what the task is intending to measure as they are completing the IAT and may even be aware of their performance. It is not known whether either of these is involved in producing the end IAT result [21]. Our respondents are self-selected and so there may be a selection bias but we do not have reason to believe that the distribution of the IAT scores will be dramatically different with a larger or very different sample. Our sample size is quite large compared to other IAT types that are often conducted under laboratory conditions with a relatively homogeneous group, such as students.

We could not control for onward dissemination of the survey link and anyone who receives the survey link could open it and be directed to take the test. Participants could, if they so choose to, take the test as many times as they wished. Nosek et al. [29] found that patterns were consistent even when multiple submissions from the same individuals were removed. The use of the Quantile regression was helpful in the fact that different characteristics seem to have different effects in different ends of the distribution. However, we note that our models generally had fairly low goodness-of-fit statistics and very few of our covariates were significant in any of our models.

## Conclusion

Stereotype activation is automatic, but stereotype application may be a controllable process and some have used strategies to counter negative country of origin effects [4]. Even though our sample, on average, exhibited implicit associations that were pro-Rich Country with respect to research quality, it does not follow that these will automatically be applied in practice, evaluating research articles for example. However, our findings from this study and previous work [17–20] certainly raises the need to assess this possibility and supports the case for further research into country-of-origin effects in research [38]. The majority of our participants associate Good Research with Rich Countries, compared to Poor Countries. Implicit associations such as these might disfavor research from poor countries in research evaluation, evidence-based medicine and diffusion of innovations.
